# Renin and electrolytes indicate the mineralocorticoid activity of fludrocortisone: a 6 year study in primary adrenal insufficiency

**DOI:** 10.1007/s40618-022-01889-1

**Published:** 2022-08-10

**Authors:** F. Ceccato, M. Torchio, I. Tizianel, M. Peleg Falb, M. Barbot, C. Sabbadin, C. Betterle, C. Scaroni

**Affiliations:** 1 Endocrinology Unit, Department of Medicine DIMED, University of Padova, Via Ospedale Civile, 105, 35128 Padua, Italy; 2 Endocrine Disease Unit, University-Hospital of Padova, Padua, Italy

**Keywords:** Primary adrenal insufficiency, Mineralocorticoid treatment, Fludrocortisone, Renin

## Abstract

**Context:**

Fludrocortisone (FC) is the mineralocorticoid (MC) replacement treatment for patients with primary adrenal insufficiency (PAI).

**Objective:**

To explore the dose of FC treatment and its relationship with glucocorticoid therapy, sodium, potassium, renin and clinical parameters.

**Setting:**

Monocentric cohort.

**Patients:**

Data of 193 patients with PAI (130 autoimmune) were collected during baseline (T0), intermediate (T1) and last follow-up visit (T2, respectively, after a mean of 38 and 72 months).

**Main outcome measure:**

Utility of endocrine and clinical parameters to titrate FC dose.

**Results:**

FC dose (50–75 μg/daily) was stable in the follow-up in half patients. The MC activity of FC was dose-dependent: we observed a reduced but significant positive linear correlation between FC dose and sodium (*r* = 0.132) and negative linear correlation between FC and potassium (*r* = − 0.162) or renin (*r* = − 0.131, all *p* < 0.01). An overall reduction in the FC dose was observed at T2 in the group with longer follow-up (> 60 months, *p* < 0.05). Higher doses of FC were observed in patients with low-normal renin, especially in autoimmune PAI (86 vs 65 μg/daily, *p* < 0.05). On the contrary, reduced sodium and increased potassium levels were observed in patients with high renin at T2. The number of cardiovascular events (15 in the whole cohort) was similar in patients sorted by renin levels or FC dose.

**Conclusions:**

Renin and electrolytes can indicate the MC activity of FC treatment: they should be routinely evaluated and used to titrate its dose that can be reduced in the long-term follow-up.

## Introduction

Primary adrenal insufficiency (PAI) is a rare (prevalence 100 to 144 per million in Western countries) and life-threatening condition, characterized by insufficient production of glucocorticoids (GCs) and mineralocorticoids (MCs), due to a primary adrenal disease [1]. The most common causes of PAI are the autoimmune adrenalitis called Addison Disease (AD, more than 50–70% of PAI in the largest series) and Congenital Adrenal Hyperplasia (CAH), followed by infectious or neoplastic bilateral conditions (tuberculosis, adrenalectomy for primary or metastatic cancer), and rare genetic causes [2–4]. GC and MC replacement treatments are lifesaving in patients with PAI.

Several short and long-acting GCs are used in patients with PAI: the former are hydrocortisone (HC, nowadays available also in the modified-release [MR-HC] formulation) and cortisone acetate (CA), the latter are prednisolone and dexamethasone (especially in CAH) [2, 5–7]. The international guidelines suggested monitoring GCs replacement using clinical assessment including body weight, postural blood pressure (BP), and signs of low or excessive GC treatment, proposing to adjust treatment with clinical response beyond endocrine evaluation. Achieving the correct replacement GC dose is a challenge, especially to identify the lowest one that relieves symptoms of insufficiency, while avoiding cortisol excess, associated with higher risk of cortisol-related complications [8, 9]. Several authors proposed serum, salivary or urinary biomarkers, as well as clinical score, but none has been extensively studied [5, 10–14].

The renin–angiotensin–aldosterone system (RAAS) is regulated with positive and negative feedbacks, and plays a crucial role in the regulation of BP, because it is one of the main drives of fluid volume status and electrolyte balance. Renin, through angiotensin I and II, stimulates aldosterone synthesis and release [15]. Free aldosterone binds the mineralocorticoid receptor (MR) in the cytosol of epithelial cells, especially in the distal nephron, resulting in increased sodium ion transport across the cell membrane [16]. Cortisol and aldosterone bind equally to the MR, the specificity of aldosterone action is provided in many tissues by the presence of 11β-hydroxysteroid dehydrogenase type 2 (11β-HSD-2), the enzyme able in inactivating cortisol to cortisone [17]. Fludrocortisone (FC, 9α-fluorocortisol) is the main MC used since its discovery in 1954: it exhibits 200 to 400-fold higher MC potency than HC and ten-fold higher than aldosterone [18]. Plasmatic peak of FC appears 2 h after oral absorption; the fluorine atom on carbon 9 protects FC from rapid conversion by 11β-HSD-2 and guarantees the access to the MR [18, 19].

The guidelines recommended monitoring MC replacement primarily based on clinical assessment and blood electrolyte measurements. General well-being, electrolytes within the normal range, and normal BP without evidence of postural hypotension indicate adequate MC replacement. Plasma renin activity in the upper normal range has been found to be a useful marker for identifying a correct MC dose [1]. A recent paper in 2020 reported the results of a web-based retrospective observational study conducted in an international cohort of patients with PAI (especially CAH, 16% of them were AD); authors concluded that routine monitoring of BP and serum electrolytes are most informative than renin concentration to titrate FC dose [20].

Nowadays, the correct titration of FC treatment is not fully established and reported in literature, making difficult to achieve in routine clinical practice the balance between under-replacement (with the risk of signs/symptoms of MC deficiency) and over-replacement (excessive activation of the MR is detrimental for the cardiovascular homeostasis, as happened in primary aldosteronism [21]).

Our aims are to explore the dose of FC treatment in a large monocentric cohort of patients with PAI in a real-life setting, to collect endocrine and biochemical data to assess whether a modification of FC is able to affect the MC balance (electrolytes, BP), and to explore if renin levels are accurate to study the activity of the RAAS.

## Materials and methods

### Patients

This retrospective study included patients with PAI evaluated at the outpatient clinic of the Endocrine Unit from September 1999 to June 2021. All patients were registered in the web-based Padova University-Hospital database, their selection has been performed using a dedicated query (ongoing GC and FC treatment in all visits, *n* = 193 patients). Specific inclusion criteria for this study were:

1. Confirmed diagnosis of PAI. A further classification consisted in
  - Autoimmune PAI (*n* = 130): characterized by positive anti-adrenal cortex or anti-21-hydroxylase antibodies. We considered isolated AD; autoimmune polyendocrine syndrome type 1 (APS-1, caused by mutations of the autoimmune regulator gene and characterized by AD associated with a various combination of other conditions, in particular chronic mucocutaneous candidiasis and/or hypoparathyroidism [22]) and other autoimmune or non-autoimmune diseases; autoimmune polyendocrine syndrome type 2 (APS-2, characterized by AD associated with autoimmune thyroid disease and/or type 1 diabetes mellitus) and autoimmune polyendocrine syndrome type 4 (APS-4, where AD was associated with other autoimmune conditions not included in APS-1 and 2) [23];
  - Bilateral adrenalectomy (BLA) for secreting or non-secreting adrenal masses (*n* = 32);
  - Salt-wasting CAH: patients with 21-hydroxylase deficiency (*n* = 20);
  - Other causes of PAI, as infection-related PAI (mainly tuberculosis), Allgrove Syndrome, x-linked adrenoleukodystrophy, mitotane-induced PAI (*n* = 11).
2. Clinical, laboratory and endocrine data available during outpatient visit. A follow-up visit was planned 3–6 months after the diagnosis in the first year, then every 6–12 months, according to clinical evaluation.

To categorize a variable follow-up of patients (from 1 to more than 20 years) and to consider a clinical-practice perspective, data collected included the baseline consultation (termed T0, the first visit after the onset of PAI), an intermediate visit (termed T1, selected as that in the middle from T0 and T2 when several consultations were available) and the last follow-up visit (termed T2).

Data collected included age of PAI onset and gender. During every outpatient visit we collect weight and height, to calculate body mass index (BMI, kg/m^2^) and body surface area (BSA, with the DuBois & DuBois formula, expressed in m^2^), systolic and diastolic BP levels (performed in the early afternoon, we reported the mean of three measurements in sitting position; postural hypotension was assessed if clinically suspected or reported), electrolytes, morning ACTH (ng/L), renin [plasma renin concentration (PRC) mIU/L, also expressed in upper limit of normality (ULN) of PRC or plasma renin activity (PRA) µg/L/h], glucose and HbA1c levels, lipid profile. Renin was not collected in case of concomitant medication that can increase its levels. From the electronic Case Report Form we retrieved current doses of GCs and FCs, cardiovascular events (acute myocardial infarction and related therapeutic procedures, stroke and transient ischemic attack, heart failure, deep vein thrombosis, pulmonary embolism, cardiac arrhythmia, peripheral occlusive arterial disease) and concomitant medications. All patients with PAI were well-educated and aware of their high-risk condition of adrenal crisis: they were advised to increase GC therapy only in the event of illness, body temperature > 38 °C, major/minor surgery, endoscopic procedures, or other events that might precipitate an adrenal crisis [1]. They were registered with a medical alert service and received an alert card, and scheduled for annual training sessions by nurses on how to manage their daily medication and any minor or moderate concurrent illnesses.

To analyze different treatments properly, we considered HC equivalent (HC Eq) doses following the proportion: 20 mg hydrocortisone HC (HC Eq = HC × 1) = 25 mg cortisone acetate CA (HC Eq = CA × 0.8) = 0.75 mg dexamethasone (HC Eq = dexamethasone × 26.67) = 5 mg prednisolone (HC Eq = Prednisolone × 4) [24].

Our study complies with the Strengthening the Reporting of Observational Studies in Epidemiology (STROBE) statement and guideline [25].

The Ethics Committee of Padova University Hospital (Comitato Etico per la Sperimentazione Scientifica) approved the study (protocol No. 80574-2021).

### Statistical analysis

Proportions and rates were calculated for categorical data. Continuous data were reported as mean and standard deviation (SD), or median and interquartile range (IQR), according to their distribution. Groups were compared with the chi-square test for categorical variables (the raw *p* values were adjusted with the Bonferroni method to take multiple comparisons into account), and with paired Student’s *t* test or Mann–Whitney test for quantitative variables. A Spearman rank-order correlation was run to assess the relationship between individual variables.

The SPSS 24 software package for Windows (SPSS, Inc., Chicago, IL, USA) was used to manage the database and perform the statistical analysis. The significance level was set at *p* < 0.05 for all tests. All data analyzed during this study are included in the data repositories of the University of Padova—Research Data UniPD [26].

## Results

### Clinical and endocrine characteristics

According to the selection criteria, 193 patients were analysed at baseline visit (T0). An intermediate visit (T1) was available 38 ± 28 months after T0 (median 35 months), a follow-up visit (T2) was collected 35 ± 20 months after T1 (median 35 months). The follow-up of patients is detailed in supplementary table 1 [26].

Clinical and biochemical data of the whole cohort in the considered visits are reported in Table 1, in supplementary Tables 2–4 [26] we reported the same data divided by aetiology of PAI. From T0 to T2, we observed an increase in glucose levels in all patients with PAI (especially those after BLA and those with salt-wasting CAH), total and LDL cholesterol levels were reduced in the whole cohort and in autoimmune PAI. Arterial hypertension was diagnosed in 8% of patients at baseline visit or during follow-up: according to guidelines, if BP levels were still increased after FC titration, a treatment with angiotensin II receptor blockers (*n* = 4) or angiotensin-converting enzyme blockers (*n* = 6) was used, combined with calcium blocker (*n* = 2).

**Table 1:** Clinical and endocrine data of patients with PAI at baseline (T0), intermediate (T1) and last visit (T2)

	T0	T1	T2
Age (years)	43 ± 1.0 (15–83)	46 ± 1.0^a^ (15–87)	49 ± 1.0^a,b^ (17–89)
Height (cm)	167.2 ± 1.1 (140.0–189.5)	167.4 ± 1.0 (140.0–193)	167.65 ± 1.0 (147.0–189.5)
Weight (Kg)	66.8 ± 1.9 (37.0–125.0)	68.9 ± 1.4 (37.0–114.5)	70.7 ± 1.5 (38.0–120.0)
BMI (Kg/m^2^)	24.57 ± 0.8 (13.98–43.25)	24.62 ± 0.6 (16.44–39.10)	25.18 ± 0.6 (16.00–38.75)
BSA (m^2^)	1.75 ± 0.03 (1.26–2.32)	1.78 ± 0.02 (1.26–2.29)	1.79 ± 0.03 (1.30–2.37)
Systolic BP (mmHg)	117 ± 2 (80–170)	121 ± 1 (85–170)	122 ± 1 (80–185)
Diastolic BP (mmHg)	78 ± 1 (50–100)	79 ± 1 (40–100)	80 ± 1 (50–115)
Na^+^ (mmol/L)	139.0 ± 0.3 (123.0–150.0)	138.8 ± 0.2 (127.0–147.0)	138.9 ± 0.2 (130.0–145.0)
K^+^ (mmol/L)	4.19 ± 0.04 (2.90–5.70)	4.18 ± 0.03 (2.90–5.70)	4.20 ± 0.0 (2.90–5.40)
ACTH (ng/L)	469.1 ± 106.6 (3.5–9580.0)	487.59 ± 109.2 (1.5–7000.0)	440.50 ± 116.2 (1.6–11,140.0)
PRC (mIU/L)	177.65 ± 39.8 (0.6–2933.0)	153.72 ± 153.7 (0.1–1459.0)	218.50 ± 47.7 (1.6–5144.0)
Glucose (mmol/L)	4.81 ± 0.2 (3.60–15.87)	4.96 ± 0.1^c^ (2.70–12.38)	5.09 ± 0.1^d^ (3.83–11.43)
HbA1c (mmol/mol)	42.49 ± 2.9 (26.00–99.00)	43.59 ± 1.7 (29.00–76.00)	42.34 ± 1.47 (26.00–76.00)
Total cholesterol (mmol/L)	5.71 ± 0.2 (2.28–11.92)	5.52 ± 0.1^e^ (3.26–8.24)	5.41 ± 0.1^f^ (2.65–9.04)
HDL cholesterol (mmol/L)	1.69 ± 0.1 (0.63–4.01)	1.71 ± 0.1 (0.68–4.72)	1.69 ± 0.1 (0.81–4.59)
LDL cholesterol (mmol/L)	3.20 ± 0.2 (0.88–9.50)	3.26 ± 0.1 (1.68–5.44)	3.06 ± 0.1^g^ (0.85–5.96)
Tryglicerides (mmol/L)	1.36 ± 0.1 (0.41–4.49)	1.42 ± 0.1 (0.32–4.19)	1.43 ± 0.1 (0.55–4.98)

Data are reported as mean ± standard error, range in brackets

*BMI* Body Mass Index, *BSA* Body Surface Area, *BP* blood pressure, *PRC* Plasma Renin Concentration, *HDL* high-density lipoprotein, *LDL* low-density lipoprotein, *HbA1c* glycosylated haemoglobin

^a^*p* < 0.001 vs T0

^b^*p* < 0.001 vs T1

^c^*p* = 0.006 vs T0

^d^*p* = 0.004 vs. T0

^e^*p* = 0.011 vs. T0

^f^*p* = 0.009 vs. T0

^g^*p* = 0.015 vs. T0

### Longitudinal follow-up of GC treatment

GC dose was lower in BLA respect to autoimmune PAI; patients with salt-wasting CAH were treated with reduced doses of GC and higher of FC respect to autoimmune PAI, their ACTH levels were lower and renin increased (Table 2). GC treatment has remained the same for 70% of patients with autoimmune PAI (from T0 to T2, mean 6 years), the others were shifted to MR-HC: at T2 most patients were treated with short-acting GC (50% CA and 40% HC). As reported in supplementary Fig. 2 [26], patients with salt-wasting CAH were treated especially with long-acting GC (dexamethasone and prednisolone) in all the visits considered (all *p* < 0.001). On the contrary, short-acting GCs (CA and HC) were offered especially to patients with autoimmune PAI, with a decreased use of CA and an increased use of HC in the last visit (*p* < 0.001).

**Table 2:** ACTH and renin levels, mineralocorticoid and glucocorticoid treatment in the groups considered in the whole longitudinal study. Levels are depicted as mean ± standard error

	ACTH (ng/L)	PRC/ULN or PRA/ULN	PRC mIU/L	FC dose (μg)	HC Eq dose (mg)
Autoimmune PAI	341.79 ± 32.49^a^	4.05 ± 0.57^b^	185.65 ± 21.9^c^	76.53 ± 2.54	32.07 ± 0.78^c^
BLA	978.80 ± 260.74	2.2 ± 0.43	93.97 ± 19.25	69.47 ± 3.18	27.46 ± 0.58
Salt-wasting CAH	175.65 ± 42.86^d,g^	7.14 ± 3.14^h^	356.21 ± 162.35^j^	83.75 ± 5.07^e,k^	15.4 ± 0.92^f,l^
Other cause of PAI	346.54 ± 84.48	4.1 ± 0.99	157.59 ± 52.13	80.47 ± 7.51	44.69 ± 3.89

*PAI* Primary adrenal insufficiency, *BLA* bilateral adrenalectomy, *CAH* congenital adrenal hyperplasia, *ULN* upper limit of normality, *PRC* plasma renin concentration, *PRA* plasma renin activity, *FC* fludrocortisone, *HC Eq* hydrocortisone equivalents

^a^*p* < 0.001 vs BLA

^b^*p* = 0.009 vs BLA

^c^*p* = 0.002 vs BLA

^d^*p* = 0.014 vs autoimmune PAI

^e^*p* = 0.037 vs autoimmune PAI

^f^*p* < 0.001 vs autoimmune PAI

^g^*p* = 0.005 vs BLA

^h^*p* = 0.012 vs BLA

^j^*p* = 0.002 vs BLA

^k^*p* = 0.016 vs BLA

^l^*p* < 0.001 vs BLA

We observed a tendency to reduce GC replacement treatment from T0 (HC Eq 32.6 mg/daily), to T1 (30.4 mg/daily) and T2 (27.5 mg/daily, respectively *p* < 0.000 T1 vs T0 and *p* < 0.001 T2 vs T1 or T0, supplementary Fig. 3 [26]).

### Longitudinal follow-up of FC treatment

**Fig. 1:**
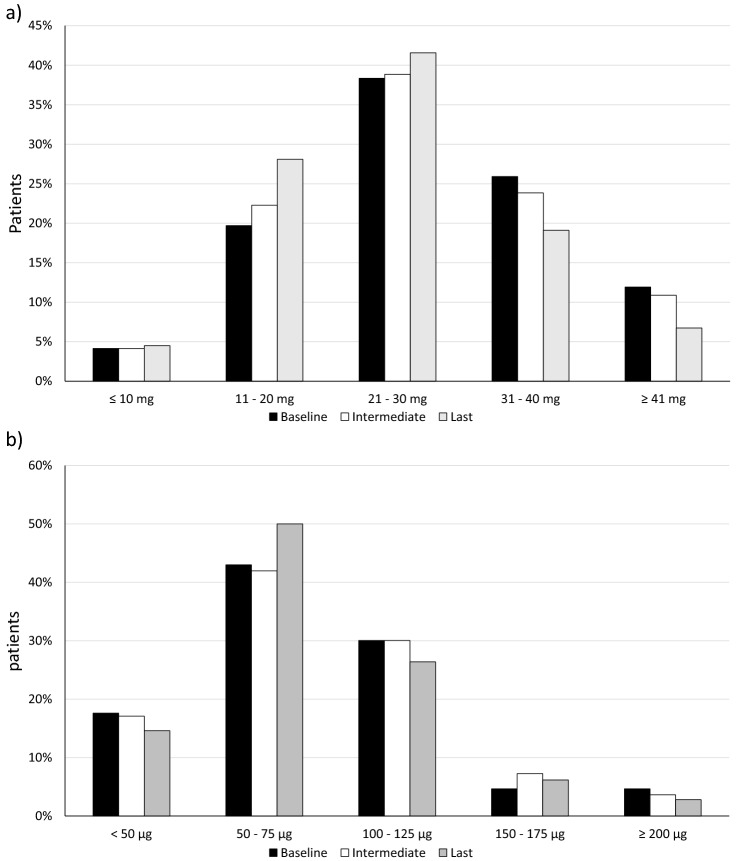
Distribution of hydrocortisone (HC, panel a) and fludrocortisone (FC, panel b) daily dose in the baseline (black bar), intermediate (white bar) and last visit (grey bar) in all patients with PAI (*n* = 193)

**Fig. 2:**
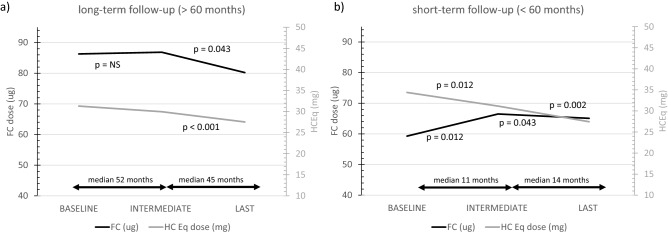
Mean daily dose of FC (black line) and HC equivalent (HC Eq, grey line) in the baseline, intermediate and last visit in patients with PAI and long-term follow up (panel **a**, 115 patients) and short-term follow-up (panel **b**, 78 patients)

Half patients were treated with 50–75 μg/daily of FC, and 50 μg was the most taken dose (corresponding to the mode of 564 visits). Less than 10% of patients were treated with high doses of FC (> 150 μg/daily, as reported in Fig. 1). In the follow-up FC dose was not changed in 131 patients (T0→T1) and 109 patients (T1→T2). FC dose was increased in 32 and 29 patients (respectively T0→T1 and T1→T2); FC dose was reduced in 30 and 40 cases (respectively T0→T1 and T1→T2). The mean FC dose remained stable if we consider the whole observation period (respectively 75.4, 78.6 and 74.9 μg/daily), also according to the different aetiologies of PAI (supplementary Fig. 3 [26]). If we divided the cohort of patients according to the length of observation period in a long-term group (> 60 months, 115 patients) and a short-term group (< 60 months, 78 patients), an increase in the FC dose was observed in the first period after diagnosis of PAI in the short-term group (Fig. 2, panel b). Nonetheless, a reduction in the FC dose was observed from intermediate to last visit in the group with a long-term follow-up (see Fig. 2, panel a).

### Renin and electrolyte levels according to FC dose in patients with PAI

**Fig. 3:**
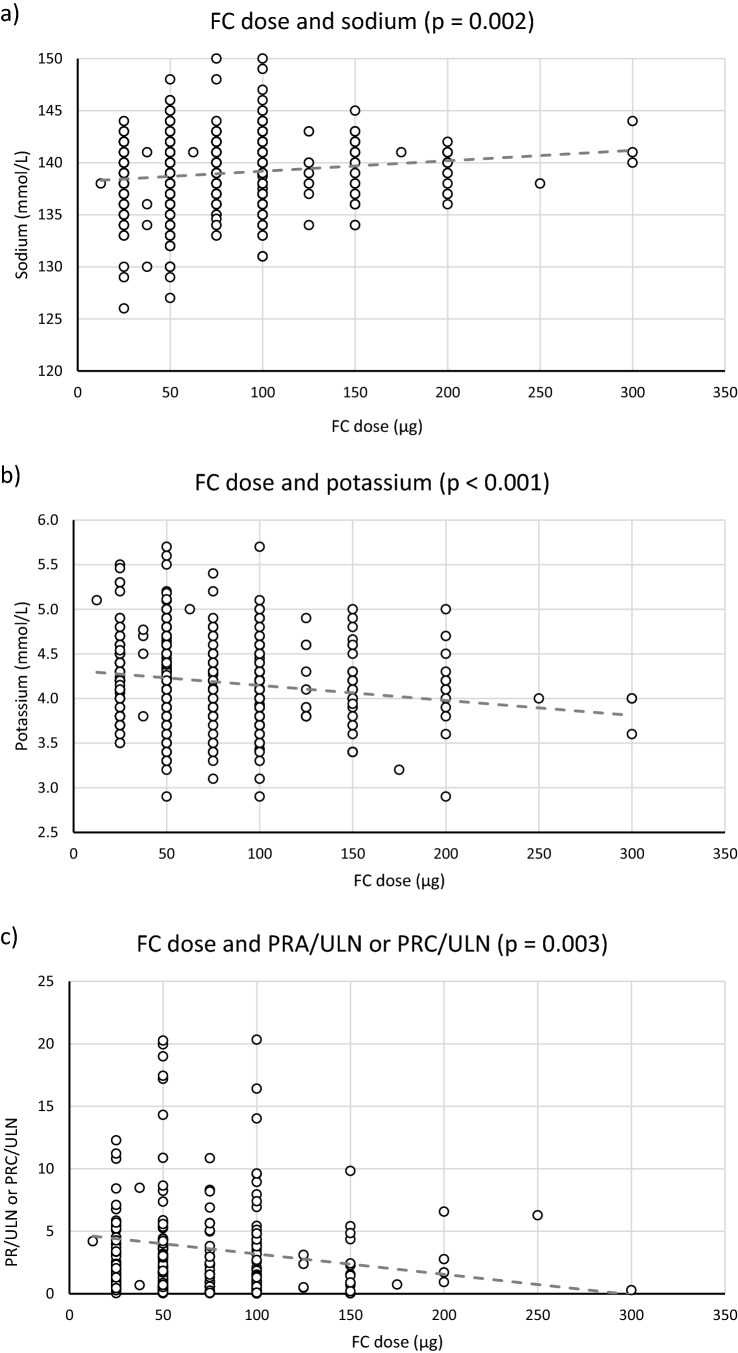
Scatter plot indicating the linear correlation between FC dose and the respective level of sodium (panel **a**), potassium (panel **b**) and the ratio PRC/ULN or PRA/ULN (panel **c**) in all visits

The MC activity of FC was dose-dependent in all visits: a positive linear correlation has been observed between FC dose and sodium levels (*r* =  + 0.132, *p* = 0.002), and negative linear correlations have been reported between FC dose and potassium (*r* = − 0.162, *p* < 0.001) or PRC levels (*r* = − 0.131, *p* = 0.018) and the ratio PRC/ULN or PRA/ULN (*r* = − 0.124, *p* = 0.003, reported in Fig. 3).

**Fig. 4:**
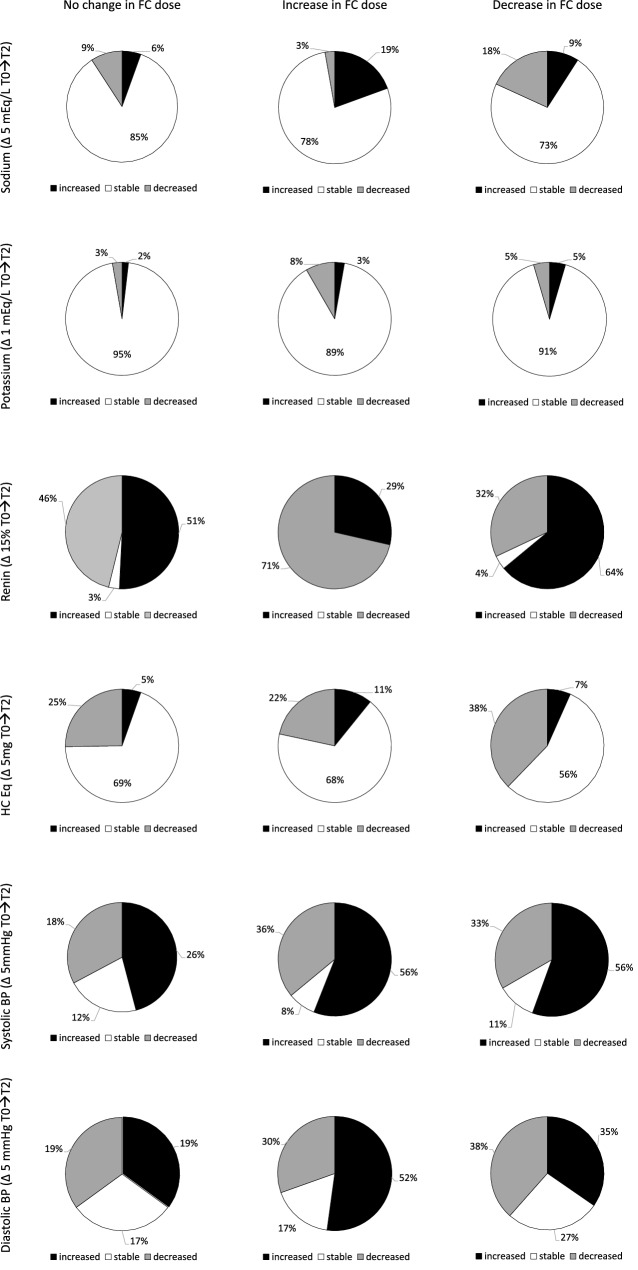
Longitudinal analysis of electrolytes, renin and HC Eq in patients with PAI at baseline and last follow-up visit (from T0 to T2, mean follow-up 73 months), according to the modification of FC treatment

In Fig. 4, we divided patients according to FC treatment (unchanged, increased or reduced from T0 to T2), and we considered changes in sodium levels (increased if Δ ≥ 5 mEq/L from baseline, reduced if Δ ≥ − 5 mEq/L, or stable), potassium levels (increased if Δ ≥ 1 mEq/L from baseline, reduced if Δ ≥ − 1 mEq/L, or stable), renin concentration (increased if Δ ≥ 15%, reduced if Δ ≥ − 15% deviation from baseline, or stable), HC Eq levels (increased if Δ > 5 mg from baseline, reduced if Δ > − 5 mg, or stable) and systolic or diastolic BP (increased if Δ ≥ 5 mmHg from baseline, reduced if Δ ≥ − 5 mmHg, or stable). In patients with increased FC dose during follow-up (*n* = 36) renin was reduced from T0 to T2 in 71% of patients. On the other hand, patients with a reduced FC treatment in the follow-up (*n* = 44) presented an increase of renin from baseline to last visit in 64% of patients. The reduction of GC replacement treatment, observed in the whole cohort of cases, was unrelated to FC dose, as electrolytes or BP levels.

Finally, we divided our cohort according to the renin levels available at T2, measured as PRC in the same laboratory (*n* = 128), and defined low-normal renin if PRC < 46.1 mIU/L (39%) and high renin if PRC > 46.1 mIU/L (61%). Reduced sodium and increased potassium levels were observed in patients with high-renin (respectively 138 vs 141 nmol/L, *p* < 0.001 and 4.3 vs 4 nmol/L, *p* = 0.002), without differences in systolic (respectively 121 vs 122 mmHg) or diastolic BP (both 79 mmHg). A tendency to use higher doses of FC was observed in patients with low-normal renin (81 vs 69 μg/daily, *p* = 0.1), being significant in autoimmune PAI (86 vs 65 μg/daily, *p* = 0.049, detailed in Table 3). The number of cardiovascular events (15 in the whole cohort) was similar in patients divided by renin levels or FC dose. In most cases the cardiovascular event can be related to other conditions: active cancer (*n* = 2), anti-phospholipid syndrome (*n* = 1) or previous history of Cushing’s syndrome (*n* = 4).

**Table 3:** Clinical and endocrine features of patients with autoimmune PAI at last visit (*n* = 84), divided according to renin levels

	Low-normal renin (< 46.1 mIU/L)	High-renin (> 46.1 mIU/L)	*p* value
Age years	47.9 ± 2.5	49.8 ± 1.6	0.527
Weight (kg)	70.4 ± 2.7	70.4 ± 2.9	0.995
Systolic BP (mmHg)	117 ± 3.3	122 ± 1.7	0.104
Diastolic BP (mmHg)	77 ± 2.2	80 ± 0.9	0.208
Fludrocortisone dose (μg/daily)	86 ± 10.8	65 ± 4.6	0.049
HC Eq dose (mg/daily)	30.4 ± 1.8	28.5 ± 1.3	0.403
Sodium (nmol/L)	140.6 ± 0.4	137.9 ± 0.4	< 0.001
Potassium (nmol/L)	3.9 ± 0.1	4.3 ± 0.1	< 0.001
ACTH (ng/L)	294.2 ± 64.9	255.4 ± 54.7	0.65
Glucose (nmol/L)	4.9 ± 0.2	5.1 ± 0.1	0.636
HbA1c (mmol/mol)	40.3 ± 2.8	41.8 ± 2.3	0.699
Total cholesterol (mmol/L)	5.5 ± 0.2	5.4 ± 0.2	0.513
HDL cholesterol (mmol/L)	1.6 ± 0.1	1.7 ± 0.1	0.692
LDL cholesterol (mmol/L)	3.3 ± 0.2	3.1 ± 0.2	0.57
Tryglicerides (mmol/L)	1.4 ± 0.1	1.2 ± 0.1	0.299
Months T0→T1	41.1 ± 4	39.4 ± 3.5	0.745
Months T1→T2	31.3 ± 2.8	30.5 ± 2.3	0.815

Data are presented as mean and standard error

*BMI* Body Mass Index, *BSA* Body Surface Area, *BP* blood pressure, *PRC* Plasma Renin Concentration, *HDL* high-density lipoprotein, *LDL* low-density lipoprotein, *HbA1c* glycosylated haemoglobin

## Discussion

GC and MC replacement treatments are lifelong required in patients with PAI. Recently, efforts have been conducted to reduce overall GC therapy to the lowest tolerated GC dose [8, 9]. On the contrary, MC replacement treatment with FC has been less evaluated and described in literature. The guidelines recommended to titrate MC replacement with clinical assessment (BP) and blood electrolyte measurements; furthermore, increased PRAin the upper normal range has been proposed [1].

A recent observational study reported that serum electrolytes are more informative than other parameters (as renin) to titrate FC dose. However, it was a multi-centre web-based study (with intrinsic bias related to the design and data collection) in a selected cohort of patients with PAI (more CAH than AD, different from the higher prevalence of autoimmune disease in the adult population with PAI), and GC dose was not considered [20]. Moreover, most of commonly-used GCs (HC, prednisolone) present a low MC activity: it is possible that the increased doses of GCs used can be used as a FC-sparing strategy [27]. As a matter of fact, renin plays a key-role in the RAAS, since it is controlled with a negative feedback by MR [16], which is activated by FC in patients with PAI [27]. To evaluate the most appropriate parameter to evaluate the correct therapy with FC, we collected data regarding MC treatment (FC dose) and MC activity (renin, electrolytes, BP) in a large monocentric cohort of patients with PAI and a regular follow-up in a referral Endocrinology Unit.

We described a cohort of patients with PAI with a majority of autoimmune AD (67%), according to clinical prevalence. We performed a longitudinal analysis between baseline, intermediate and follow-up-visits: the mean time span in the selected period was respectively 38 and 73 months from the first visit (achieving a mean observation period of 6 years,  > 12 years in some patients). Before our study, the previously longitudinal assessment of FC treatment in PAI described a median time between assessments of 433 days (without an intermediate visit, and the longer follow-up lasted less than 6 years) [20]. Our observation period, close to a study recently reported in patients with adrenal insufficiency [28], is sufficient to propose a modification in the replacement treatment and consistent to observe a measurable clinical-endocrine difference. A long-term follow-up is of utmost importance because the modification of a lifelong treatment, to personalize replacement therapy, requires several attempts and a close doctor-patient relationship to increase treatment adherence and reduce therapeutic inertia [29].

Overall, ACTH levels were lower in patients with CAH despite they did not use a higher dose of GCs. As a matter of fact, long-acting GC are more used in patients with CAH to suppress the ACTH-mediated synthesis of steroid precursors [2]. On the contrary, FC dose used was higher in salt-wasting CAH, probably to offset the reduced GC treatment (and the absent RAAS activity of dexamethasone). ACTH levels were higher in patients after BLA, despite GC dose: patients with AD or CAH may present a residual cortisol secretion [30], which is completely cleared by adrenalectomy. BLA was performed in 8 patients with Cushing’s disease: 4 developed a Nelson’s syndrome with a significant increase in size of the pituitary adenoma [31].

Short-acting GCs are the most used, as expected [32], and CA is the preferred in Italy, because HC is not immediately available and needs specific prescription by an endocrinologist [28]. CA and HC treatment was used respectively in 50% and in 40% of the cohort at the last follow-up visit (20% direct-release HC and 20% MR-HC), especially in patients with autoimmune PAI. CA is characterized by a lower MC effect than HC, and overall HC eq dose was > 30 mg/day only in 25% of the entire cohort: it is possible that the effect of GC dose in renin and electrolytes is reduced. According to the interplay between GC and FC, a prospective and controlled study can pave the way to novel strategy of treatment. Through follow-up, a general reduction of GC has been reported (mean decrease of 16%), according to the modern concept of reducing hormonal treatment to prevent cardiovascular and skeletal cortisol-related comorbidities [28, 32, 33]. The observed reduction in total and LDL cholesterol in the longitudinal evaluation can reflect both an increased use of lipid lowering medication in the aging patients and the reduction of GC treatment, as observed in hypopituitary patients [34].

In our cohort, the mean FC dose remained stable in all different types of PAI if the observation period was short (median 35 months, up to 5 years), with a small increase soon after the baseline visit (11 months from T0 to T1). We can speculate it is an attempt of the endocrinologist to relief some residual symptoms of PAI, early after its onset. On the contrary, in those patients with a long-term follow-up, the dose of FC in the intermediate visit was much the same at the baseline (median time 52 months), and we observed a reduction of FC from the intermediate to the last visit (after mean 45 months), without reporting signs or symptoms of adrenal insufficiency. It seems that it took several years to propose minimal modifications of FC replacement treatment. In authors’ opinion, the main limitation of MC treatment titration is the formulation itself, available in 100 μg tablet: it is difficult to obtain lower doses, as 12.5 μg. Moreover, a reduction of FC requires time for being proposed and established: a significant FC reduction from intermediate to last visit was observed only in patients with a long-term follow-up.

Renin levels are a marker of RAAS and are controlled by several factors (sodium intake, plasma volume, age and so on). The FC treatment presents MC activity in our cohort, because with high FC doses we observed an excessive activation of the MR. According to univariate analysis, the correlation between FC and electrolytes was weak but significant: it was positive with sodium (increased sodium with high FC dose) and negative with potassium (reduced potassium with high FC dose). Therefore, FC presents an aldosterone-like effect on the MR, with activation of the Na^+^-K^+^-ATPase (the final player of the RAAS). Accordingly, renin levels decreased with higher FC dose, therefore not only electrolytes, but also renin is influenced by FC treatment. 50–75 μg/daily of FC was the most used doses, lower than that recently reported (90% of autoimmune AD with 100–200 μg/daily and 50% of salt-wasting-CAH with 100 μg/daily) [20].

Considering renin levels at the last visit, 61% of subjects presented with an increased PRC: they were characterized by reduced sodium and increased potassium levels (both in the range of normality). The increase in renin levels from baseline to the last visit is secondary to MC treatment, because aging in healthy normotensive humans induces a reduction of plasma renin levels [35]. In the whole cohort, FC dose remained stable in half cohort of patients with PAI during the observation period. Modification of FC treatment from baseline to last visit affected RAAS according to the MC activity of FC: we reported an increased renin in those patients that reduced FC, and a reduced renin in those who increased FC dose. These changes were not sufficient to induce a clinical modification in the electrolyte balance, probably because sodium and potassium levels are influenced also by GC treatment and dietary intake in PAI. Our data contrast to a recent real world data [20]: we can speculate that our lower FC dose presented and the population of PAI were sufficient to unveil the relationship between renin and FC dose. During the follow-up, an increase or a decrease in systolic or diastolic BP appear unrelated to FC titration. In authors’ opinion, there are several confounding factors that affect BP in patients with PAI. First, the GC treatment and its modification during follow-up: we observe an overall HC Eq reduction, which can result in the improvement of BP levels, as previously described [36]. On the other hand, BP increases with aging, especially systolic levels [37]. In our cohort a modification of FC dose was able to modify renin without a detrimental effect on electrolyte balance and BP levels. Therefore, we suggest to reconsider renin, combined with serum electrolytes, in the routine monitoring of MC treatment in patients with PAI. We can speculate that renin should be measured to exclude its suppression (or levels in the lower quartile of normality), as ACTH in titrating GC treatment. This could be helpful in patients with increased cardiovascular risk. However, changes of MC dose could also affect several cognitive and emotional functions, considering the complex role of MR and GR in neuronal activity [38]: for example, verbal memory improved significantly during high MR occupation [39]. Other studies are needed to support our hypothesis, especially to confirm that the titration of replacement therapy should be based upon several clinical and endocrine parameters.

Despite strengths, our study does have limitations. It is a retrospective analysis; however, data are collected in the same referral Endocrinology Unit, thus limiting selection and treatment bias. The follow-up visits were done at different time points; nonetheless, we put an effort to consider three visits (first-intermediate-last) considering that our patients present with different follow-up. Extensive medical records were available in many patients; nonetheless, a prospective study is more accurate than an observational one. Titration of FC dose presents an intrinsic limitation due to its 100 μg formulation: in our opinion, an effort of the healthcare companies could be the conduction of a prospective and controlled clinical trial with different FC tablets, with a pre-established algorithm to modify FC dose according to clinical/endocrine criteria rather than physician preference, also assessing compliance to treatment.

In conclusion, in our long-term observational study, we report that FC dose can be modified in the follow-up of patients with PAI, as the recent efforts in the endocrine community to reduce GC treatment. Those patients treated with normal-high doses of FC can be subjected to a partial RAAS activation (reduced renin, reduced potassium and increased sodium levels) with a possible increase in cardiovascular risk. On the contrary, a rise in renin levels is observed in those patients with PAI underwent to a reduction of FC dose. BP levels are controlled by several clinical, endocrine and dietary factors: the modification of FC dose alone is not able to modify them. The replacement therapy in adult patients with PAI is complex and depending by the balance of GCs and FCs. To evaluate their optimal combination, in addition to the clinical assessment, renin and electrolytes should be considered as markers to titrate FC treatment.

## Data Availability

All data generated or analyzed during this study are included in this published article or in the data repositories listed in the references.
